# cGAS inhibitor IMSB301 modifies interferon signalling in peripheral mononuclear cells of *SAMHD1* genetic interferonopathy *in vitro*

**DOI:** 10.1002/cti2.70090

**Published:** 2026-03-19

**Authors:** Velda X Han, Jessica P Hayes, Lijun Sun, Teresa Mooneyham, Michelle Lorentzos, Hiroya Nishida, Markus J Hofer, Brian Gloss, Ruwani Dissanayake, Xianzhong Lau, Shekeeb S Mohammad, Shrujna Patel, Russell C Dale

**Affiliations:** ^1^ Khoo Teck Puat‐National University Children's Medical Institute, National University Health System Singapore Singapore; ^2^ Department of Paediatrics National University Singapore Singapore Singapore; ^3^ Kids Neuroscience Centre, The Children's Hospital at Westmead, Faculty of Medicine and Health, University of Sydney Sydney NSW Australia; ^4^ ImmuneSensor Therapeutics Dallas TX USA; ^5^ Kids Advanced Therapeutics, Kids Research, Sydney Children's Hospitals Network Sydney NSW Australia; ^6^ School of Medical Sciences and Discipline of Child and Adolescent Health, Faculty of Medicine and Health The University of Sydney Sydney NSW Australia; ^7^ Department of Brain & Neurosciences Tokyo Metropolitan Institute of Medical Science Tokyo Japan; ^8^ School of Life and Environmental Sciences and Charles Perkins Centre, Faculty of Science University of Sydney Sydney NSW Australia; ^9^ Westmead Research Hub, Westmead Institute for Medical Research Westmead NSW Australia; ^10^ Australian Genome Research Facility Ltd Westmead NSW Australia; ^11^ Australian Genome Research Facility Ltd Melbourne VIC Australia; ^12^ The Children's Hospital at Westmead Clinical School, Faculty of Medicine and Health, University of Sydney Sydney NSW Australia

**Keywords:** Aicardi–Goutières syndrome, cGAS‐STING pathway, neuroinflammation, therapeutics

## Abstract

**Objectives:**

Aicardi–Goutières syndrome (AGS) is a rare genetic interferonopathy because of aberrant DNA or RNA metabolism that lacks effective disease modifying therapies.

**Methods:**

Single‐cell RNA sequencing was performed on peripheral blood mononuclear cells (PBMCs) obtained from a patient with AGS because of pathogenic biallelic *SAMHD1* variants to assess baseline gene dysregulation compared to an age‐ and sex‐matched control. The patient and control's PBMCs were incubated with the novel clinical stage cGAS inhibitor IMSB301 for 24 h, followed by evaluation of its effects on *in vitro* gene expression.

**Results:**

In PBMCs from the patient with *SAMHD1* mutation, at baseline, the most upregulated enriched pathways were ‘response to virus’ and ‘response to type 1 interferon’; these were also the most downregulated pathways after *in vitro* IMSB301 treatment. The top five most upregulated genes at baseline were interferon‐stimulated genes (ISG) *IFIT1, IFIT3, IFI44L, ISG15, OAS1*, which were downregulated to control levels after *in vitro* treatment with IMSB301.

**Conclusion:**

The cGAS inhibitor IMSB301 resulted in specific reduction in interferon signalling *in vitro* in PBMCs from a patient with *SAMHD1* mutation, indicating a potential therapeutic role in genetic interferonopathy.

## Graphical abstract

Depicts the pathways and mechanisms of the drug.

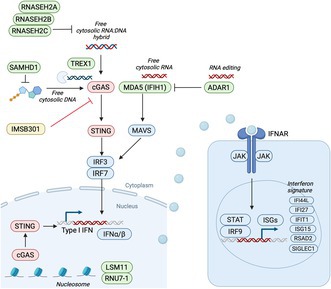

## Introduction

**Figure 1:**
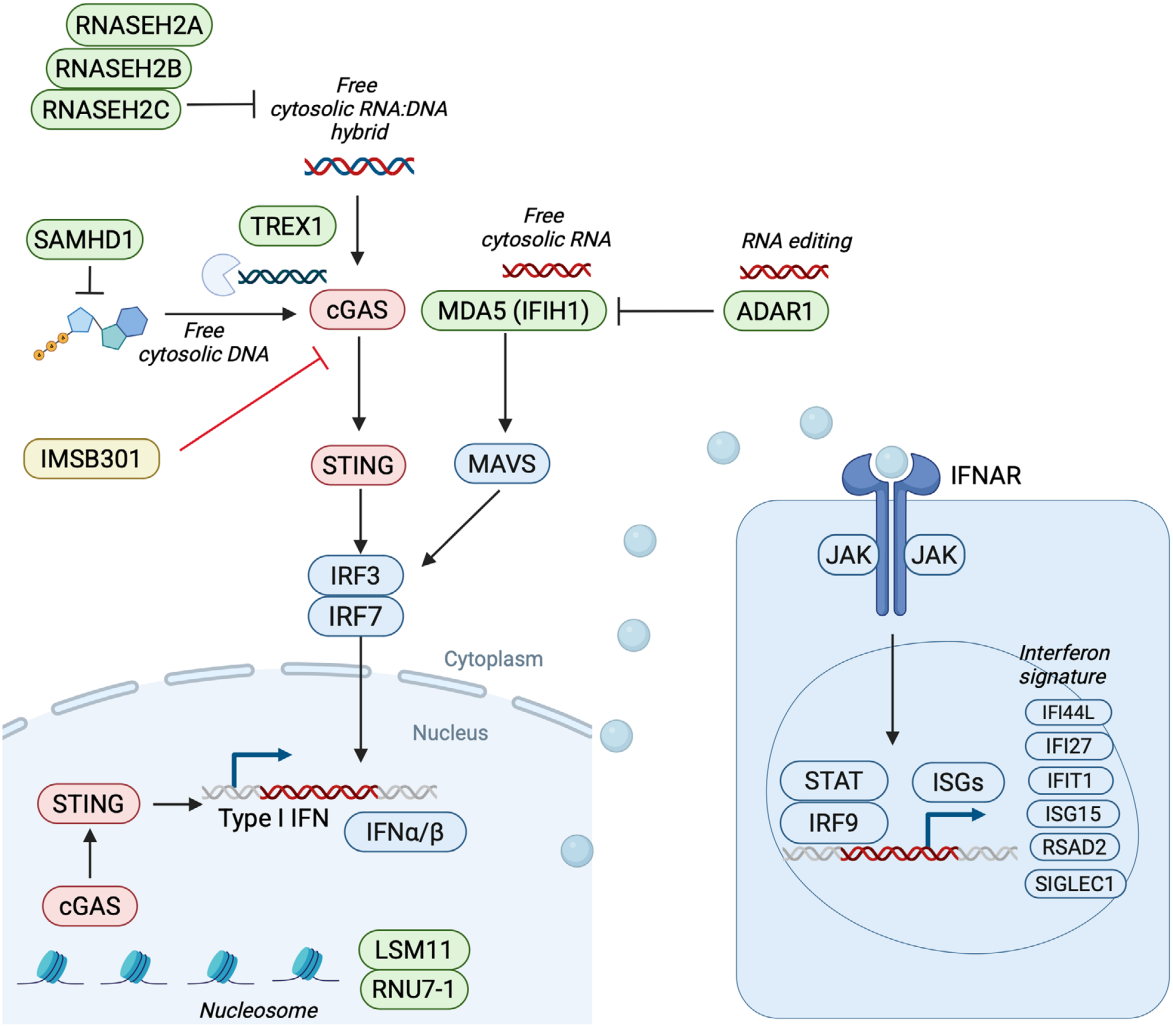
IMSB301 inhibits cGAS‐STING signalling pathway. Mutations in AGS genes such as *SAMHD1* disrupt normal nucleic acid metabolism, leading to accumulation of cytosolic DNA (other AGS genes are also presented: *TREX1, RNASEH2A, RNASEH2B, RNASEH2C, LSM11* and *RNU7‐1* which act via the cGAS signalling pathway). Elevated cytosolic dsDNA activates the cGAS‐STING pathway, which in turn triggers downstream JAK‐STAT signalling and upregulates interferon‐stimulated genes (ISGs), driving chronic antiviral responses. A six‐gene interferon signature, comprising the ISGs *IFI27, IFI44L, IFIT1, ISG15, RSAD2* and *SIGLEC1*, is used to quantify type I interferon activity in individuals with AGS. Pharmacological inhibition of the cGAS‐STING pathway may reduce this aberrant interferon/ISG activation and downstream inflammation. *ADAR* and *IFIH1* are other AGS genes, which act via the *MAVS* signalling pathway.

Aicardi–Goutières syndrome (AGS) is a rare, genetically mediated interferonopathy caused by mutations in genes such as *SAMHD1, RNASEH2A/B/C, TREX1, ADAR1*, *IFIH1*, *LSM11* and *RNU7‐1*, that leads to early‐onset neuroinflammation, cerebral calcifications and progressive neurologic impairment.^1^, ^2^, ^3^ These mutations disrupt nucleic acid metabolism, resulting in chronic activation of the innate immune system (Figure 1). This persistent immune activation drives aberrant type I interferon signalling via the Janus kinase (JAK)‐signal transducer and activator of transcription (STAT) pathway, leading to upregulation of interferon‐stimulated genes (ISGs) including *IFI27, IFI44L, IFIT1, ISG15, RSAD2* and *SIGLEC1*, which form a six‐gene ‘interferon signature’ biomarker in AGS (Figure 1).^4^

JAK inhibitors have been trialled in AGS and may improve cutaneous features, but neurological disease is not substantially improved by these therapies.^5^ In addition, toxicity and side effects pose major hurdles for standard use of JAK inhibitors in AGS.^6^ This highlights the need to develop more effective treatments that target the disease pathway. Emerging therapies that target the ‘cyclic GMP‐AMP synthase’–‘stimulator of interferon genes’ (cGAS–STING) pathway, which senses cytosolic DNA and triggers type I interferon responses, offer a more specific upstream therapeutic approach than JAK inhibitors. cGAS activation triggers cGAMP production, STING signalling, inflammation and tissue damage.^7^, ^8^, ^9^ The cGAS‐STING pathway is a key driver of AGS,^10^ and its inhibition could more directly suppress the aberrant innate immune activation underlying disease pathogenesis (Figure 1).

We investigated the *in vitro* effects of a novel cGAS‐STING inhibitor IMSB301 using peripheral blood mononuclear cells (PBMCs) from an individual with AGS and age‐ and sex‐matched control. We employed single‐cell RNA sequencing (scRNA‐seq) to characterise the baseline transcriptional signature and assess the changes induced by IMSB301 treatment.

## Results

### Baseline and IMSB301 treatment of SAMHD1 PBMC

**Figure 2:**
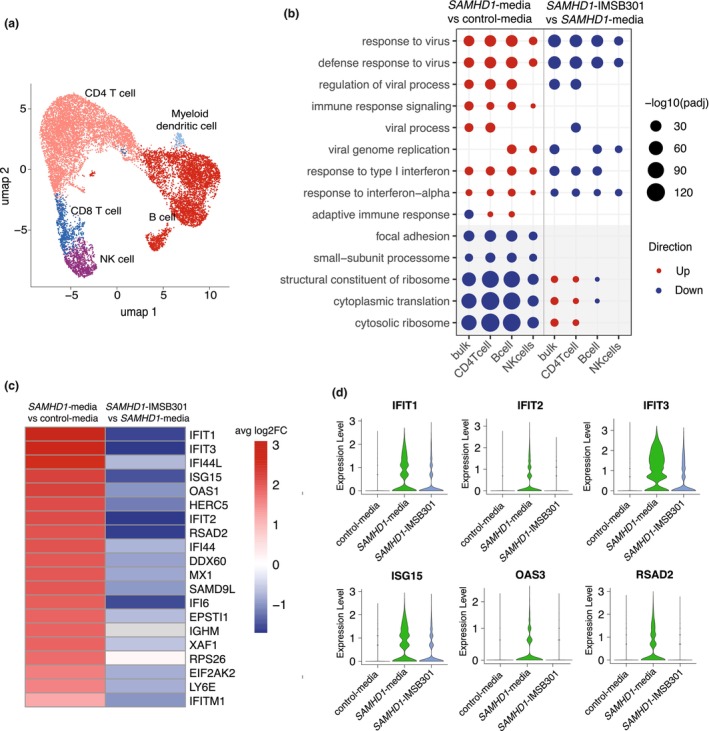
Single‐cell RNA sequencing (scRNA‐seq) of *SAMHD1*‐media vs control‐media and *SAMHD1‐*IMSB301 vs *SAMHD1*‐media. **(a)** Uniform manifold approximation and projection (UMAP) analysis of samples revealed five distinct cell clusters including B cells, CD4 T cells, CD8 T cells, natural killer (NK) cells and myeloid dendritic (mDC) cells. **(b)** Dot plot of top five up‐ and downregulated GO pathways per cell type. In the *SAMHD1*‐media vs control‐media comparison (left), top upregulated GO pathways (in red) included ‘response to virus’, ‘defense response to virus’ and ‘type I interferon signalling’, while top downregulated GO pathways (in blue) included ‘cytosolic ribosome’ and ‘cytoplasmic translation’. Following IMSB301 treatment (right), baseline antiviral and interferon pathways were downregulated, whereas ribosomal and translation pathways were upregulated. The size of the dot represents −log_10_(p.adjust). This dot plot includes only pathways involving at least three cell types across comparisons. CD8 T cells and mDCs were excluded because of the low number of cells and therefore low number of significant pathways. **(c)** Heatmap of the top 20 upregulated DEGs in bulk analysis of scRNA‐seq at baseline (*SAMHD1*‐media vs control‐media, on the left). The DEGs were predominantly interferon‐stimulated genes (ISGs), including *IFIT1, IFIT3, IFI44L, ISG15, OAS1, IFIT2, RSAD2, MX1* and *IFITM1* (ranked by fold change). These ISGs were predominantly downregulated following *in vitro* IMSB301 treatment (right). Red and blue indicate up‐ and downregulation, respectively, based on average log_2_ fold change (avg log_2_FC). **(d)** Violin plots showing the gene expression of ISGs (*IFIT1, IFIT2, IFIT3, ISG15, OAS3, RSAD2*) in control‐media, *SAMHD1*‐media and *SAMHD1*‐IMSB301 conditions from bulk analysis of scRNA‐seq. The width of the violin plot represents the density of cells with that expression level.

A total of 13 244 cells were sequenced across the samples. IMSB301 had no significant effect on the percentage of mitochondrial transcripts compared with *SAMHD1*‐media (Supplementary figure 1). Uniform manifold approximation and projection (UMAP) analysis of samples revealed five distinct cell clusters including B cells, CD4 T cells, CD8 T cells, natural killer (NK) cells and myeloid dendritic (mDC) cells (Figure 2a). In the *SAMHD1*‐media vs. control‐media comparison, bulk analysis of scRNA‐seq identified 593 upregulated and 833 downregulated differentially expressed genes (DEGs) (FDR < 0.05), with cell‐type‐specific DEGs ranging from 11 to 1426 (Supplementary figure 1). In the *SAMHD1*‐IMSB301 vs. *SAMHD1*‐media comparison, bulk analysis of scRNA‐seq identified 232 upregulated and 94 downregulated DEGs (FDR < 0.05), with cell‐type specific DEGs ranging from 0 to 154.

### Enriched pathways across cell types in SAMHD1‐media versus control‐media, and after IMSB301 treatment

In *SAMHD1*‐media vs. control‐media, the top five upregulated GO pathways in bulk and across all cell types included ‘response to virus’, ‘defense response to virus’ and ‘response to type 1 interferon’ signalling. The top downregulated GO pathways across all cell types included ‘cytosolic ribosome’ and ‘cytoplasmic translation’ (dot plot in Figure 2b, all pathways in Supplementary data 1, bar charts of top 10 pathways in Supplementary figure 2). CD8 T cells and myeloid dendritic cells made up only a small fraction of the total cell types and generated few significant pathways, so were excluded from the dot plot.

After IMSB301 treatment (*SAMHD1*‐IMSB301 vs. *SAMHD1*‐media), the baseline GO pathways, which were upregulated at baseline, including ‘response to virus’ and ‘type I interferon signalling’, were downregulated (Figure 2b). Conversely, GO pathways that were downregulated at baseline, such as ‘cytosolic ribosome’ and ‘cytoplasmic translation’, were upregulated following IMSB301 treatment (Figure 2b).

### Reversal of differentially expressed genes within ‘Response to virus’ pathway by IMSB301 treatment

We further examined the DEGs within the top GO pathway, ‘response to virus,’ identified from the bulk analysis scRNA‐seq data. This pathway was upregulated at baseline (*SAMHD1*‐media vs. control‐media) but showed the most significant downregulation following IMSB301 treatment (*SAMHD1*‐IMSB301 vs *SAMHD1*‐media). Within this pathway, 43 genes were upregulated at baseline and downregulated after IMSB301 treatment. The 43 genes were grouped into three functional categories, including cytosolic and endosomal nucleic acid sensors, IFN signalling and transcriptional regulators, and effector interferon‐stimulated genes (Supplementary table 1).

### Top differentially expressed genes upregulated at baseline in SAMHD1‐media, and downregulated after *in vitro* IMSB301 treatment

The top 20 most significantly upregulated DEGs in bulk analysis of scRNA‐seq data at baseline (*SAMHD1*‐media vs control‐media) were predominantly ISGs (Figure 2c, ranked by fold change in the heatmap), including *IFIT1, IFIT3, IFI44L, ISG15, OAS1, IFIT2, RSAD2, MX1 and IFITM1* (adj *P*‐value = 10^−176^ to 10^−305^). These genes were predominantly downregulated following *in vitro* treatment with IMSB301 (*adj P*‐value = 10^−42^ to 10^−293^).

Average expression of representative genes in bulk analysis of scRNA‐seq, including *IFIT1, IFIT2, IFIT3, ISG15*, *OAS3* and *RSAD2* are illustrated using violin plots (Figure 2d). These plots show upregulation of the genes in *SAMHD1‐*media vs control‐media, and downregulation following *in vitro* IMSB301 treatment, with some genes showing expression levels comparable with control levels.

In contrast, *in vitro* IMSB301 treatment of healthy control cells resulted in only six downregulated DEGs, with lower log fold changes and lower *P*‐values (Supplementary table 2). Of these six DEGs, only one gene, *XAF1*, is an interferon‐related gene.

## Discussion

In this study, analysis of blood scRNA‐seq from *SAMHD1*‐deficient cells compared with healthy control cells revealed broad upregulation of viral response pathways, type I interferon signalling and an interferon signature, along with downregulation of ribosomal and mRNA‐binding pathways. These alterations were reversed following *in vitro* treatment with the novel cGAS‐STING inhibitor IMSB301, with key interferon genes showing marked downregulation and normalisation to control levels. We identified similar findings across all cell types. These findings highlight the ability of cGAS inhibition to reduce aberrant interferon‐driven transcriptional programs and demonstrate its potential as an effective therapeutic strategy for AGS.

The current therapeutic approach in AGS involves targeting type I interferon signalling, with JAK inhibitors and reverse transcriptase inhibitors being the most studied options.^5^, ^11^ JAK inhibitors, particularly baricitinib, reduce interferon‐stimulated gene expression and may improve some neurological symptoms, though responses vary and cytopenias or transaminitis can occur.^11^ Other JAK inhibitors have uncertain efficacy.^12^ Reverse transcriptase inhibitors (abacavir, lamivudine and zidovudine) show transient biomarker improvement without sustained IFN‐α reduction or clear clinical benefit.^13^ Traditional immunosuppressants (steroids, IVIG) have yielded inconsistent results thus far.^12^ Although JAK inhibitors have been shown to modulate interferon activity and improve systemic features of AGS, they act downstream in the pathway (Figure 1), have limited effect on established neurological damage because of poor central nervous system penetration, delayed diagnosis and irreversible injury.^5^, ^11^, ^13^ This highlights the urgent need for more effective therapies in AGS.

Based on our *in vitro* experiment, cGAS‐STING inhibition appears to be effective using IMSB301. cGAS inhibition dampens core JAK‐STAT and interferon regulatory factor (IRF)‐mediated interferon signalling, reducing downstream antiviral ISG expression and antiviral response. Pattern‐recognition receptors detect cytosolic or endosomal DNA resulting in interferon signalling driven by cGAS‐STING; when cGAS is inhibited, reduction in upstream DNA sensing suppresses transcription of interferon‐stimulated genes. Consequently, cGAS inhibitors are expected to exert therapeutic benefits in AGS patients. IMSB301 is currently in clinical development, progressing from a completed Phase 1a study in healthy volunteers to an upcoming Phase 1b trial in patients (ISRCTN90049550).

cGAS‐STING pathway inhibitors are being explored beyond AGS in conditions driven by chronic innate immune activation. In autoimmune diseases such as systemic lupus erythematosus, these inhibitors aim to reduce aberrant interferon signalling and inflammation, with compounds advancing through early clinical trials.^14^ In neurodegenerative disorders including Alzheimer's and Parkinson's disease, targeting cGAS‐STING may help mitigate neuroinflammation.^15^ Although IMSB301 has low brain penetrance, future development of brain‐penetrant cGAS inhibitors will be important for addressing neurological conditions.

A limitation of this study is the single patient and control, and examination of only *SAMHD1‐associated* AGS. A further limitation is the use of PBMCs, which are dominated by lymphocytes, with low numbers of innate immune cells. Future studies should include testing of samples beyond *SAMHD1*, human clinical trials with accompanying *ex‐vivo* scRNA seq testing to couple biological with clinical effects in genetic interferonopathies.^16^, ^17^

## Conclusion

Single‐cell RNA sequencing revealed broad viral response and type I interferon activation in an individual with *SAMHD1*‐mutation, which was reversed following *in vitro* treatment with a novel cGAS inhibitor, IMSB301. This case highlights the potential of scRNA‐seq for understanding disease mechanisms and therapeutic responses in rare, single‐patient (*n* = 1) studies.^18^ Furthermore, these findings provide support for the development of cGAS inhibition as a therapeutic strategy for AGS and related interferonopathies.

## Methods

### Individual with AGS and control

We recruited one adult with genetically confirmed AGS. This was a 22‐year‐old male carrying pathogenic compound heterozygote mutations in exon 4 of *SAMHD1* (c.433C > T/p.R145X and c.490C > T/p.R164X), with confirmed parental segregation. He presented at 2 years of age with a progressive dystonic and spastic motor syndrome lasting 3 years, accompanied by mild intellectual disability. His neurological picture has remained static for the past 12 years; he is without aids and suffers chilblains throughout the winter. Healthy control was an age‐ and sex‐matched 25‐year‐old male.

### Blood collection

Venous blood from the participants was collected in Vacutainer ACD tubes (BD Biosciences, BD367756) after gaining written consent for this study. Peripheral blood mononuclear cells (PBMCs) were isolated and stored, as previously described.^19^ None of the participants were acutely unwell with infections two weeks prior to blood taking. None of the participants have ever taken immune therapies.

### *In vitro* treatment of PBMCs with IMSB301

Frozen PBMC aliquots were thawed rapidly in a 37°C water bath, followed by washing with thawing medium consisting of RPMI 1640 supplemented with 10% foetal bovine serum (FBS), 1% GlutaMAX™ and 1% HEPES. The cells were then incubated in culture medium (RPMI 1640 supplemented with 10% FBS and 1% GlutaMAX™) at 37°C in a 5% CO_2_ atmosphere for 3 h. I*MSB301 is a novel, orally available small molecule* designed to specifically inhibit the cGAS enzyme. Following the incubation, the cells were treated with 2 μM IMSB301 (Immune Sensor) in culture media or control culture media (untreated) for 24 h at 37°C in a 5% CO_2_ atmosphere. IMSB301 inhibited DNA‐induced IFN‐β production in human PBMCs with an IC_50_ of 0.24 μM. For this study in AGS PBMCs, we selected a concentration 8–10 times above the IC_50_ to ensure sufficient inhibition.

### HIVE™ single‐cell RNA‐sequencing

PBMCs from an individual with *SAMHD1*‐associated AGS and one control were treated with IMSB301. In total, four HIVE devices were used: an individual with AGS (*SAMHD1*‐media and *SAMHD1*‐IMSB301 treatment) and an age‐sex matched control (control‐media and control‐IMSB301). PBMCs were incubated, loaded into HIVE devices with capture beads and processed for single‐cell NGS library preparation using established protocols (Supplementary data 2).^18^, ^20^ Following transcript capture and library completion (AGRF Ltd), final libraries were quality‐checked and sequenced on an Illumina® NovaSeq® X system.

### Single‐cell RNA sequencing bioinformatic analysis

ScRNA‐seq data were analysed in the R statistical environment with *tidyverse*. For HIVE scRNA‐seq, the *Seurat* package was used for analysis. Cells with a high mitochondrial transcript ratio (> 0.15) were excluded. Experiments were integrated using the *FindIntegrationAnchors* function in *Seurat*; then, immune cell types were assigned using *scPred*. Merged data were then split by cell type and separately normalised, scaled and integrated between patients using *harmony;* then, UMAP (uniform manifold approximation and *projection)* projections were made using the first 30 dimensions. Differentially expressed genes were identified using *FindMarkers*.

### Over representation pathway analyses

Enriched pathways were identified based on over representation analysis (ORA) to obtain Gene Ontology (GO) pathways, based on the false discovery rate (FDR). Significant GO pathways (FDR < 0.05) were further simplified using the *simplify* function in *clusterProfiler*.

### Other graphs

Dot plots of GO results were plotted using the *ggplot2* package. The top five upregulated and downregulated GSEA GO post‐simplified pathways for each cell type were selected as representative GO terms. Only those GO terms present in more than two cell types in the pre‐simplified GSEA GO pathway list were mapped. The heatmap of individual genes' log_2_FC (fold changes) and violin plots of individual genes' expression were plotted using the *ggplot2* package.

### Ethics statement

Ethics approval was granted by the Sydney Children's Hospitals Network Human Research Ethics Committee (2021/ETH00356).

## Author Contributions

RCD and JPH conceptualised and designed the experiments. RCD recruited the patients. JH, RD, XL performed the experiment. BG and VXH performed bioinformatic analysis. VXH, JPH, LS, ML, HN, SP, SSM, RCD analysed and interpreted the data. VXH and RCD wrote the initial draft. All authors critically revised and edited the manuscript. All authors approved the final version of the article.

## Conflicts of interest

Lijun Sun and Teresa Mooneyham are employees of ImmuneSensor Therapeutics and receive stock in the company. Dr. Sun is a co‐inventor of US patents (10947206 and 12091387) covering cGAS inhibitors.

## Conflict of interest

The authors declare no conflict of interest.

## Funding

ImmuneSensor Therapeutics.

## Disclosure

We acknowledge the use of OpenAI's ChatGPT (model: GPT‐5; OpenAI, San Francisco, USA) to assist in the generation of Tables. ChatGPT was used to group genes identified by bioinformatic analysis into functional subclusters and to generate concise descriptions of gene functions. All outputs were edited and verified by authors for scientific accuracy and validity prior to inclusion in the manuscript.

## Supporting information


Supplementary data 1

Supplementary figures 1–2

Supplementary data 2

Supplementary tables 1–2

## Data Availability

Deidentified data can be requested by any qualified investigator by contacting russell.dale@sydney.edu.au.
