# Supplementary material for: cGAS inhibitor IMSB301 modifies interferon signalling in peripheral mononuclear cells of SAMHD1 genetic interferonopathy in vitro
Source: Clin Transl Immunology. 2026 Mar 19;15(3):e70090. doi: 10.1002/cti2.70090 (PMC13093634; doi:10.1002/cti2.70090)
Supplement: Supplementary file 2 — Supplementary figures 1–2 [file CTI2-15-e70090-s004.pptx]

## Slide 1
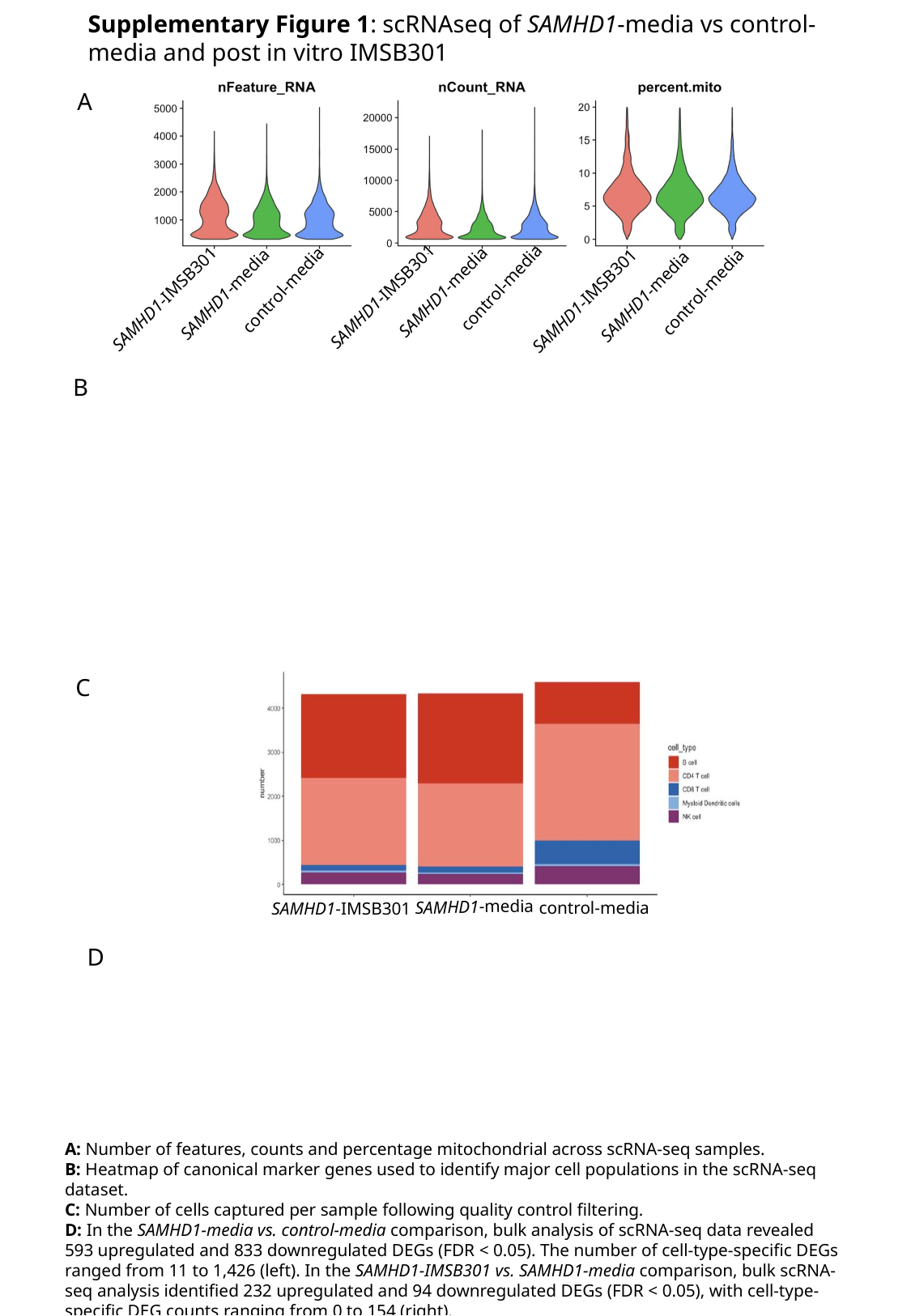

Supplementary Figure 1: scRNAseq of SAMHD1-media vs control-media and post in vitro IMSB301
A
control-media
control-media
control-media
SAMHD1-media
SAMHD1-media
SAMHD1-media
SAMHD1-IMSB301
SAMHD1-IMSB301
SAMHD1-IMSB301
B
C
D
SAMHD1-media
control-media
SAMHD1-IMSB301
D
A: Number of features, counts and percentage mitochondrial across scRNA-seq samples.
B: Heatmap of canonical marker genes used to identify major cell populations in the scRNA-seq dataset.
C: Number of cells captured per sample following quality control filtering.
D: In the SAMHD1-media vs. control-media comparison, bulk analysis of scRNA-seq data revealed 593 upregulated and 833 downregulated DEGs (FDR < 0.05). The number of cell-type-specific DEGs ranged from 11 to 1,426 (left). In the SAMHD1-IMSB301 vs. SAMHD1-media comparison, bulk scRNA-seq analysis identified 232 upregulated and 94 downregulated DEGs (FDR < 0.05), with cell-type-specific DEG counts ranging from 0 to 154 (right).

## Slide 2
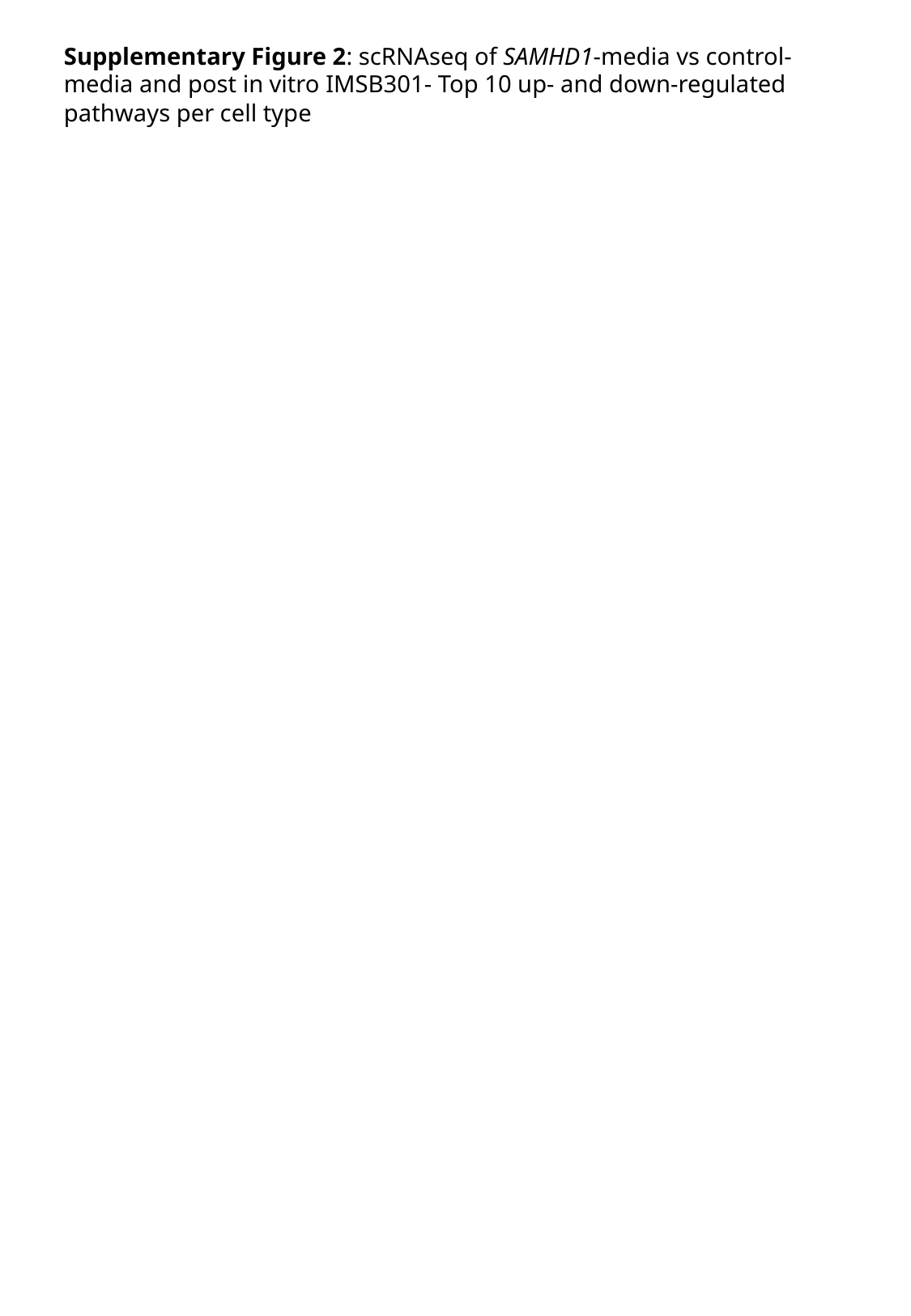

Supplementary Figure 2: scRNAseq of SAMHD1-media vs control-media and post in vitro IMSB301- Top 10 up- and down-regulated pathways per cell type
