# Supplementary material for: cGAS inhibitor IMSB301 modifies interferon signalling in peripheral mononuclear cells of SAMHD1 genetic interferonopathy in vitro
Source: Clin Transl Immunology. 2026 Mar 19;15(3):e70090. doi: 10.1002/cti2.70090 (PMC13093634; doi:10.1002/cti2.70090)
Supplement: Supplementary file 3 — Supplementary data 2 [file CTI2-15-e70090-s002.docx]

**Supplementary Methods**

HIVE™ *Single cell RNA-sequencing*

After incubation with IMSB301 or control media, approximately 30,000 PBMCs per sample were resuspended in 1 mL of DPBS supplemented with 1% FBS, and then combined with 3 mL of cell media (DPBS + 1% FBS). Single cells were allowed to settle into the picowells of the HIVE^TM^, each containing 3’ transcript-capture beads. The HIVE^TM^ devices were placed on a spin plate and centrifuged at 30 x g for 3 minutes. Following centrifugation, the media was removed, and 2 mL of sample wash solution was added to each device. After removing the wash solution, the cell-loaded HIVE^TM^ devices were frozen at -80°C with 2 mL of cell preservation solution added, prior to transfer to the Australian Genome Research Foundation (AGRF) for downstream processing and single-cell NGS library preparation (<https://honeycombbio.zendesk.com/hc/en-us/articles/15173728631707-HIVE-CLX-Sample-Capture-User-Protocol>).

According to the standard protocol, the cell-loaded HIVE^TM^ devices were sealed with a semi-permeable membrane, which enabled the application of a strong lysis solution followed by hybridisation solution. Beads with captured transcripts were extracted from the HIVE^TM^ device via centrifugation. Subsequent library preparation steps were performed in a 96-well plate format (<https://honeycombbio.zendesk.com/hc/en-us/articles/15173667166491-HIVE-CLX-Transcriptome-Recovery-Library-Preparation-User-Protocol>). The size distribution and quality of the final libraries were assessed using a TapeStation 2150 with the D5000 ScreenTape System (Agilent Technologies, Santa Clara, CA, USA), and the concentration of pooled libraries was measured via qPCR. HIVE^TM^ scRNA-seq libraries were sequenced on an Illumina® NovaSeq® X system using kit-specific primers.
