# Supplementary material for: cGAS inhibitor IMSB301 modifies interferon signalling in peripheral mononuclear cells of SAMHD1 genetic interferonopathy in vitro
Source: Clin Transl Immunology. 2026 Mar 19;15(3):e70090. doi: 10.1002/cti2.70090 (PMC13093634; doi:10.1002/cti2.70090)
Supplement: Supplementary file 4 — Supplementary tables 1–2 [file CTI2-15-e70090-s001.docx]

**Supplementary Table 1:** Differentially expressed genes from the top GO pathway ‘response to virus’ via bulk analysis of single cell RNA sequencing that were significantly (FDR) upregulated at baseline and significantly downregulated by IMSB301 treatment.

| Function | Genes | Relevance to cGAS–STING Inhibition |
| --- | --- | --- |
| Cytosolic and Endosomal Nucleic Acid Sensing | *IFI16, RIGI (DDX58), IFIH1 (MDA5), ADAR, ZBP1, DHX58, DDX60, DDX60L, TLR7* | These pattern-recognition receptors detect cytosolic or endosomal DNA/RNA. Their expression depends on IFN signalling driven by cGAS–STING. When cGAS is inhibited, loss of upstream DNA sensing suppresses their transcription. |
| IFN Signalling and Transcriptional Regulation | *IRF7, STAT1, STAT2, EIF2AK2 (PKR), USP18, NMI, PARP9, DTX3L, TRIM22, TRIM25* | Core JAK–STAT and IRF regulators mediating the interferon transcriptional response. Their downregulation reflects dampened type I IFN signalling and reduced positive feedback through IFNAR after cGAS inhibition. |
| Effector Interferon-Stimulated Genes (ISGs) | *IFI27, IFI6, IFI44, IFI44L, ISG15, ISG20, IFIT1, IFIT2, IFIT3, IFIT5, IFITM1, OAS1, OAS2, OAS3, OASL, MX1, MX2, RSAD2, BST2, HERC5, PLSCR1, LGALS9, RTP4, SHFL, NT5C3A* | Downstream antiviral and immune-regulatory ISGs induced by the IFNAR–STAT1/2 axis. Their suppression confirms that cGAS inhibition blocks STING-dependent IFN production and downstream ISG activation |

Abbreviations: cGAS, [cyclic GMP-AMP synthase](https://www.google.com/search?q=cyclic+GMP-AMP+synthase&client=firefox-b-d&sca_esv=73b20ffd545e0371&ei=f9EeaYvSLueKvr0Pitbk6AY&ved=2ahUKEwjc27WPqICRAxUimq8BHUVxE0AQgK4QegQIARAC&uact=5&oq=cGAS-STING+stands+for&gs_lp=Egxnd3Mtd2l6LXNlcnAiFWNHQVMtU1RJTkcgc3RhbmRzIGZvcjIKEAAYsAMY1gQYRzIKEAAYsAMY1gQYRzIKEAAYsAMY1gQYRzIKEAAYsAMY1gQYRzIKEAAYsAMY1gQYRzIKEAAYsAMY1gQYRzIKEAAYsAMY1gQYRzIKEAAYsAMY1gQYR0iQEFD1AVjnDnABeAGQAQCYAfwCoAHQEqoBBTItNC40uAEDyAEA-AEBmAIBoAIomAMAiAYBkAYIkgcBMaAHsiSyBwC4BwDCBwM0LTHIBxk&sclient=gws-wiz-serp&mstk=AUtExfCjxdcc0PoTNhtn1pKGW0cpTQ_dPya5TG3TotCa85E8cB-sJM7KsZBFcISJF3dD4w6USUmsN8L3baitwrf6Z6uZykFmhvBEen6LoyApNgQXm5hLrFJ1WRw74N8HxKx96LM&csui=3); IFN, interferon; IFNAR, interferon-α/β receptor; ISG, interferon stimulated genes; JAK, janus kinase; STAT, signal transducer and activator of transcription; STING, [stimulator of interferon genes](https://www.google.com/search?q=stimulator+of+interferon+genes&client=firefox-b-d&sca_esv=73b20ffd545e0371&ei=f9EeaYvSLueKvr0Pitbk6AY&ved=2ahUKEwjc27WPqICRAxUimq8BHUVxE0AQgK4QegQIARAD&uact=5&oq=cGAS-STING+stands+for&gs_lp=Egxnd3Mtd2l6LXNlcnAiFWNHQVMtU1RJTkcgc3RhbmRzIGZvcjIKEAAYsAMY1gQYRzIKEAAYsAMY1gQYRzIKEAAYsAMY1gQYRzIKEAAYsAMY1gQYRzIKEAAYsAMY1gQYRzIKEAAYsAMY1gQYRzIKEAAYsAMY1gQYRzIKEAAYsAMY1gQYR0iQEFD1AVjnDnABeAGQAQCYAfwCoAHQEqoBBTItNC40uAEDyAEA-AEBmAIBoAIomAMAiAYBkAYIkgcBMaAHsiSyBwC4BwDCBwM0LTHIBxk&sclient=gws-wiz-serp&mstk=AUtExfCjxdcc0PoTNhtn1pKGW0cpTQ_dPya5TG3TotCa85E8cB-sJM7KsZBFcISJF3dD4w6USUmsN8L3baitwrf6Z6uZykFmhvBEen6LoyApNgQXm5hLrFJ1WRw74N8HxKx96LM&csui=3).

**Supplementary Table 2:** Top downregulated differentially expressed genes (DEGs) in control-IMSB301 vs control-media comparison

| Gene | p.adjust | Average log2FC | Gene name | Gene function | Interferon- stimulated gene (ISG) |
| --- | --- | --- | --- | --- | --- |
| *PGGHG* | 3.85 E-04 | -0.53 | Peptidylglycine alpha-amidating monooxygenase pseudogene | Pseudogene, possible regulatory role in gene expression |  |
| *MALAT1* | 5.63 E-04 | -0.11 | Metastasis Associated Lung Adenocarcinoma Transcript 1 | lncRNA, regulates gene expression and cancer metastasis |  |
| *SNORD13* | 1.60 E-03 | -1.76 | Small Nucleolar RNA, C/D Box 13 | rRNA modification and processing |  |
| *KIFC2* | 9.76 E-03 | -0.84 | Kinesin Family Member C2 | Microtubule motor protein |  |
| *ARHGEF1* | 3.64 E-02 | -0.27 | Rho Guanine Nucleotide Exchange Factor 1 | Activates Rho GTPases, regulates cytoskeleton and cell movement |  |
| *XAF1* | 3.70 E-02 | -0.36 | XIAP Associated Factor 1 | Pro-apoptotic protein, regulate antiviral and interferon signalling | Yes |

Abbreviations: GTPases, guanosine triphosphatase; lncRNA, long non-coding RNA; RNA, ribonucleic acid; rRNA, ribosomal RNA; XIAP, X-linked inhibitor of apoptosis
